# Section Curve Reconstruction and Mean-Camber Curve Extraction of a Point-Sampled Blade Surface

**DOI:** 10.1371/journal.pone.0115471

**Published:** 2014-12-31

**Authors:** Wen-long Li, He Xie, Qi-dong Li, Li-ping Zhou, Zhou-ping Yin

**Affiliations:** State Key Laboratory of Digital Manufacturing Equipment and Technology, School of Mechanical Science & Engineering, Huazhong University of Science and Technology, Wuhan, P. R. China; Xiamen University, China

## Abstract

The blade is one of the most critical parts of an aviation engine, and a small change in the blade geometry may significantly affect the dynamics performance of the aviation engine. Rapid advancements in 3D scanning techniques have enabled the inspection of the blade shape using a dense and accurate point cloud. This paper proposes a new method to achieving two common tasks in blade inspection: section curve reconstruction and mean-camber curve extraction with the representation of a point cloud. The mathematical morphology is expanded and applied to restrain the effect of the measuring defects and generate an ordered sequence of 2D measured points in the section plane. Then, the energy and distance are minimized to iteratively smoothen the measured points, approximate the section curve and extract the mean-camber curve. In addition, a turbine blade is machined and scanned to observe the curvature variation, energy variation and approximation error, which demonstrates the availability of the proposed method. The proposed method is simple to implement and can be applied in aviation casting-blade finish inspection, large forging-blade allowance inspection and visual-guided robot grinding localization.

## Introduction

Blades, which include turbine blades, compressor blades, and propeller blades, are the most critical parts of an aviation engine. They work under high temperatures and pressures, and a small change in the blade geometry can affect the operation performance of the aviation engine. For quality assurance purposes, high-precision measuring techniques are used to evaluate the dimensional error of aviation blades. These techniques can be categorized into two main groups: contact measurement (coordinate measurement machine) and non-contact measurement (laser/optical scanners, X-ray and CT). A coordinate measurement machine (CMM) is equipped with a contact probe while scanning a freeform surface. The CMM is the most popular measuring method in industrial settings and has a high accuracy (1–3 µm). The low measuring speed and potential collision/interference with parts are the main concerns. With the advent of high-resolution sensors, non-contact measuring techniques, such as Breuckmann stereoSCAN 3D-HE, achieve scanning accuracies as high as 10 µm and can be used to inspect a blade during its manufacturing process (casting, forming and robot polishing).

**Figure 1:**
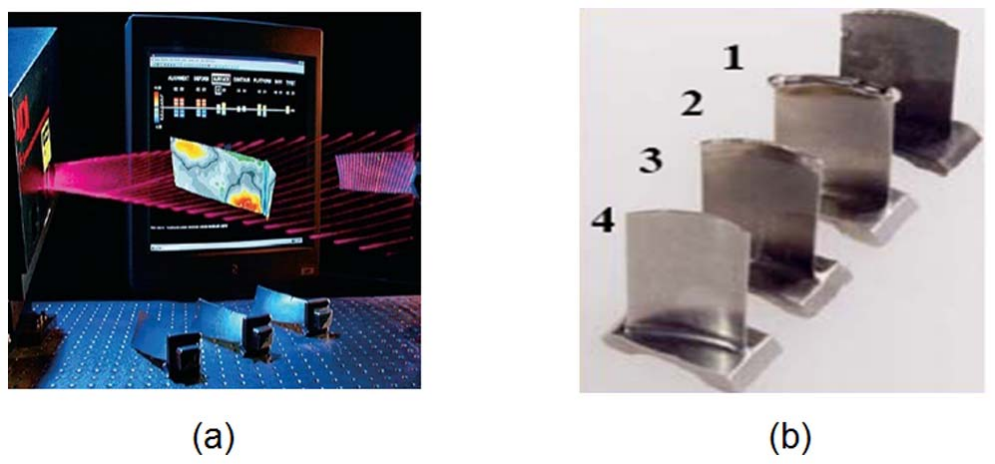
Industrial application of blade measuring. (a) Inspection (GE), (b) Repairing (MTU).

Many efforts have been made in blade inspection and repairing using both contact and non-contact measuring techniques to obtain the optimum operation performance and reliability for aviation engines. In Fig. 1, 1) **Inspection**: a blade is designed with a thin wall, a crankle surface and a difficult-to-cut material. Geometric deformation is a common problem during the manufacturing process, and it must evaluate the dimensional error of the part. The inspection and analysis of the blade section serve as an important link in blade manufacturing; 2) **Repairing**: an aviation engine is extremely expensive (costing approximately $9 million), and blade manufacturing accounts for a large proportion of this cost. Because of the high temperature and impact load, used blades may have various defects, such as wear, impact dents and cracks. The manufacturing cost associated with repairing a defected blade is as low as one third of the cost of replacing a blade. Some blade manufacturers (MTU, BCT) have begun to perform this business. The main tasks are to locate the damaged area, calculate the breakage volume and evaluate the repaired quality.

### Blade inspection

Hsu et al. [1] proposed an iterative localization algorithm for airfoil blade inspection. A CMM was used to measure the blade object, and the traditional 3-2-1 rule was applied to establish a coordinate system. A typical feature of this method is its iterative process incorporating the CMM measurement and coordinate upgrading procedure. Shortly thereafter, the same research group systematically introduced a section inspection and analysis technique for aviation blades [2]. A two-step measuring procedure was used to adapt sharp regions, such as the leading/trailing edges. The design parameters of the blade section were evaluated after an efficient localization between the measured data and their nominal curve. Chang and Lin [3] proposed an automatic blade inspection technique using a 3-axis CMM probe with a 2-axis driving head. Interference from the undercut surfaces and traveling paths were particularly focused. Makem et al. [4] presented a virtual inspection system to localize the data and evaluate the manufacturing accuracy of the aviation blade, where the errors of the root/mid/tip section thickness were inspected and analyzed. Unlike the 3-2-1 rule, the Iterative Closest Point (ICP) algorithm [5] was used to provide significantly better registration results between the point cloud and its nominal model. Savio et al. [6] presented a state of metrology of freeform shapes, which focused on the introduction of measuring techniques and related metrological issues. A blade, which is regarded as a typical freeform surface, was cited to analyze the challenging tasks in sampling, alignment and error evaluation. Heo et al. [7] presented a computer-aided measuring technique for an impeller using a CMM based on the ruled line of the CAD model, which could partition the blade surface into several unit measuring regions. To recover the surface shape of the manufacturing part from the nominal curve and measured points, Li and Ni [8] proposed an iterative method of non-rigid registration and section profile reconstruction.

Recently, Breuckmann and GE companies [9]–[10] also developed a blade inspection system based on the non-contact measuring technique and successfully applied it to blade inspection. The Breuckmann 3D inspection system operates based on an adapted structured light projector, and customized software was developed for the inspection requirement. By using the latest state-of-the-art inspection technology of Breuckmann, GE replaced the conventional CMM measuring approach with a highly time-saving and cost-saving control procedure, which significantly reduced the inspection time and generated more conclusive and easily transferrable measurement results. The non-contact measurement points are large-scale, unordered and noisy. In this situation, performing surface alignment and parameter extraction of the blade surface is not easy. Over the past few years, some iterative alignment methods for the blade surface have been developed, such as the improved ICP algorithm [11], SDM algorithm [12], ADF algorithm [13]; however, avoiding the local optimal alignment problem and accurately extracting the blade section parameters from the discrete point cloud remain a challenging task.

### Blade repairing

The repair of aviation components, particularly the blade, is a highly competitive market and actively supported by well-known engine producers, such as Rolls-Royce, MTU Aero Engines, SNECMA Moteurs and General Electric. Currently, the blade repairing processes are manually performed, but they are labor intensive and time consuming, and the quality is inconsistent because curved blades are overly complex for manual treatment. Recently, many practical studies of blade repair have been conducted by researchers at the University of Nottingham. Yilmaz et al. [14] presented the state of the research on machining and repairing turbo-machinery components. Two important repair steps were introduced and discussed: milling tool path generation and robot belt grinding/polishing. Gao et al. [15] and Yilmaz and Grindy [16] also published important studies on blade repairing based on the reverse-engineering technique. In their repair process, a CAD model was used to generate motion paths of laser welding and NC machining. Because used blades typically suffer from wear, cracks and other defects, non-contact measuring sensors were applied to locate the defecting regions. Similar to Gao et al., Zheng et al. [17] used the reverse-engineering technique to digitize the blade surface, perform point-to-surface alignment, identify the worn area and undamaged area, and generate a laser welding procedure. Berger et al. [18] proposed an integrated process for the multi-axis milling of aviation hard-cutting materials, such as titanium and nickel alloys, which is applied to the intelligent equipment and control of the ESPRIT project. Rong et al. [19] proposed a deformable-template-based method to recover the blade surface from section profiles. This method can automatically deform the nominal curve to best fit the measured points of a blade section.

Robotic grinding/polishing has attracted considerable attention in blade repairing due to its advantages of automation, flexible contact and width-line machining. Zhang et al. [20] proposed a local grinding model to simulate robot belt grinding, particularly for free-form surfaces, such as blades. Huang et al. [21] reported a successful development of an automated SMART robotic system to grind/polish vane airfoils. The system layout, section profile fitting, robot path planning and tool wear compensation during the repairing process were introduced. Chen et al. [22] proposed a rail-free robot scheme in on-site welding repair for hydraulic turbine blades with large-scale surfaces. Real-time images of the blade state from a CCD camera were used to determine the damaged area; therefore, the operator could control the robot system to perform the repair. Researchers at the ABB Corporate Research Center in Shanghai [23] briefly reviewed the robotic techniques in industrial application and noted that force control and machine vision were enabling technologies of robotic automation.

Because of its high speed and simple operation, non-contact measurement is widely applied in blade manufacturing (in addition to medical orthopedics [24] and surgical changing assessment [25]), and point cloud has received increasing attention as a representation of the blade model. The motivation of this study is to improve section curve reconstruction and mean-camber curve extraction in blade inspection and repairing. This paper introduces a new method of section curve reconstruction and mean-camber extraction. One important characteristic of this method is that both energy minimization and distance minimization are used to smooth the point cloud and generate the curve to improve reconstruction accuracy and parameter extraction accuracy as quickly as possible. First, the mathematical morphology (MM) is expanded from image processing to point cloud processing with the objective of restraining the effect of measuring defects, such as holes, uneven density and miss-registration, generating an ordered sequence of 2D measured points, and providing an initial value for the point cloud smoothing and curve reconstruction process. Next, the energy and distance are minimized to iteratively smoothen the measured points, approximate the section curve and extract the mean-camber parameter of the blade surface.

The remainder of the paper is organized as follows. Section 2 introduces an implementation of the MM operation to a point cloud, an energy minimization process for smoothing, and a distance minimization process for curve reconstruction. Section 3 introduces the mean-camber curve extraction process via the distance minimization method. Section 4 presents the experiments and analysis. Section 5 contains our conclusion and discusses the potential applications of the proposed method.

## Curve Reconstruction Based on Distance Function Minimization

### Mathematical morphology operation to a point cloud

The development and application of mathematical morphology (MM) [26], [27] originates from image processing and pattern recognition. The computation of MM is a simple combination of adding and subtracting operations based on a defined structure element. Assuming that 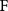 is a binary image and 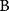 is a structure element to perform the MM operation, the dilation and corrosion can be described by
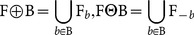
(1)


**Figure 2:**
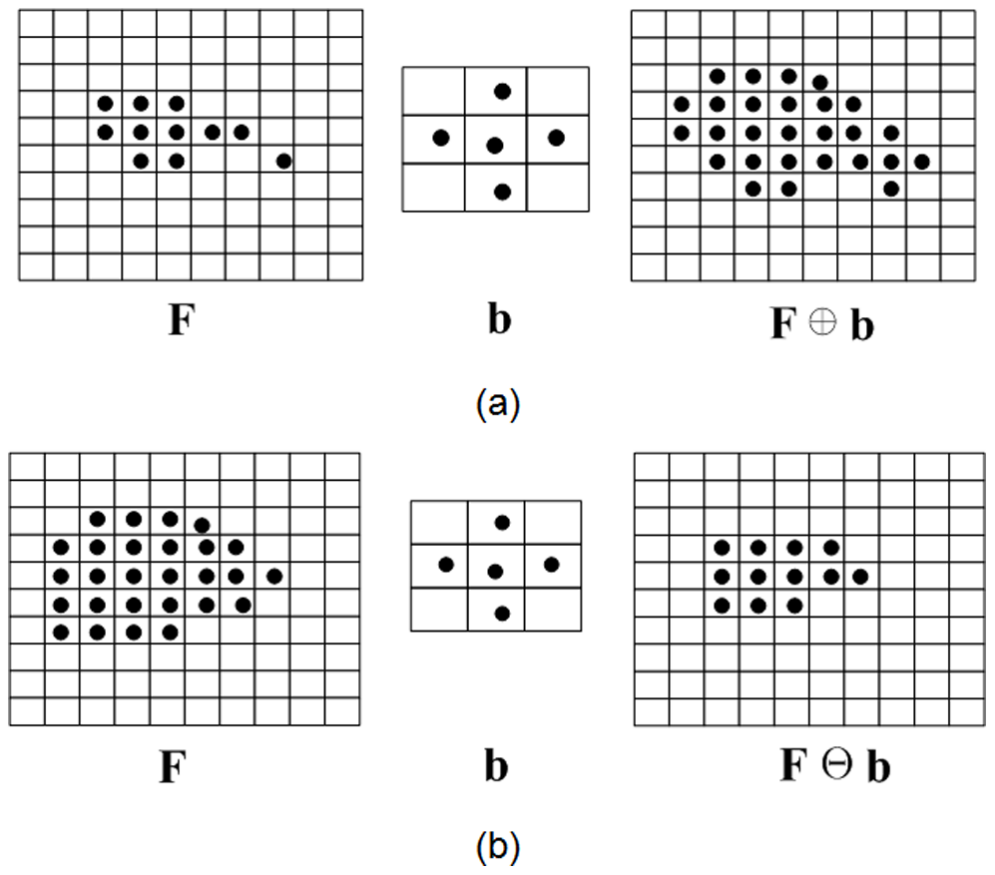
Mathematical morphology operation of a binary image. (a) dilation, (b) corrosion.

The MM operation is illustrated in Fig. 2. The dilation operation can enlarge the objective and compress the hole regions, and the corrosion operation can control overlapping regions and uneven-density regions. In the following, the MM operation is expanded from a 2D image to 3D measured points. The main objectives are to construct a well-organized point set in a section plane of the blade and provide a fast computation method of the initial value for point cloud smoothing, section curve reconstruction and mean-camber curve extraction.

The execution object of the MM algorithm is a binary image, and it should first interpret the measured points into 3D grids. Assume that the measured points from the blade model are described by 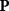 and that the mean value of sampling resolution is 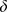. The extrema of the XYZ coordinates from 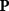 are defined as 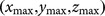 and 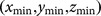. Then, the numbers of grids along the XYZ directions are
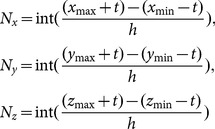
(2)where 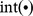 is an integer operation, 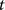 is an allowance (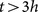), and 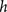 (equal to 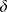) is the width of the 3D grids. For 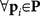, the position in the 3D grids is calculated as
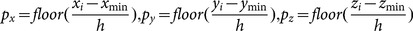
(3)where 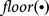 is a floor integer operation. If there is a measured point in the grid 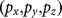, define the value of grid 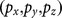 as “1”; otherwise, define it as “0”. Therefore, all measured points of 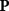 can be described using a binary image.

**Figure 3:**
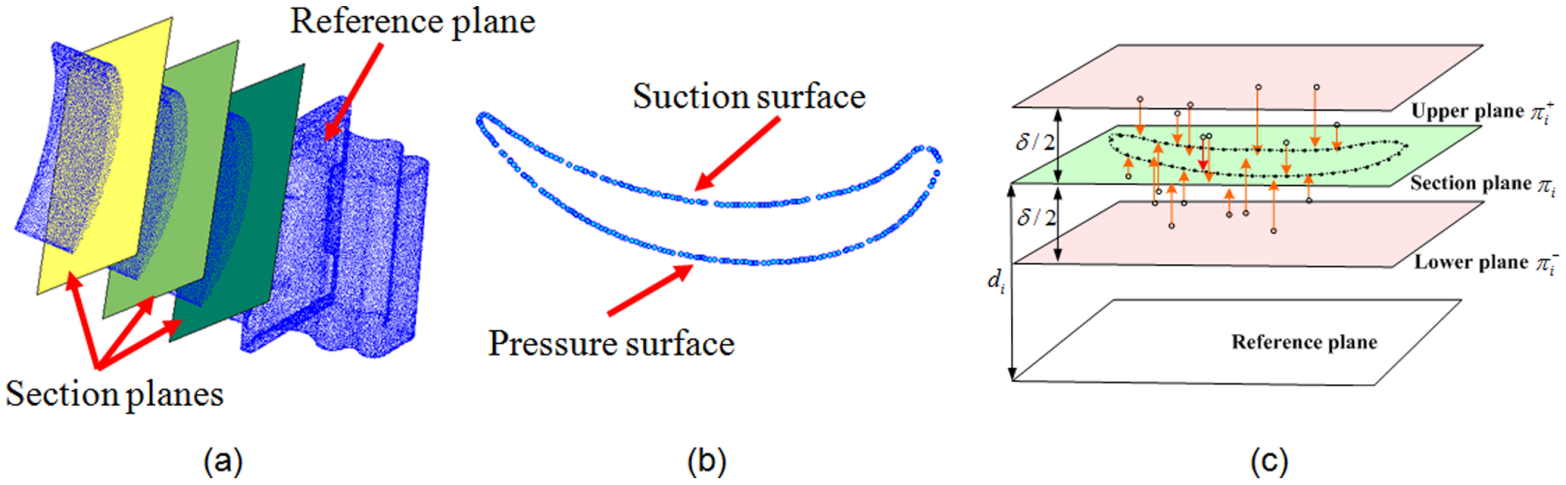
Searching for projecting points by section planes. (a) initial 3D measured points and the reference/section planes, (b) measured points in one section plane, (c) search for projecting points in a section plane 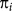.

**Figure 4:**
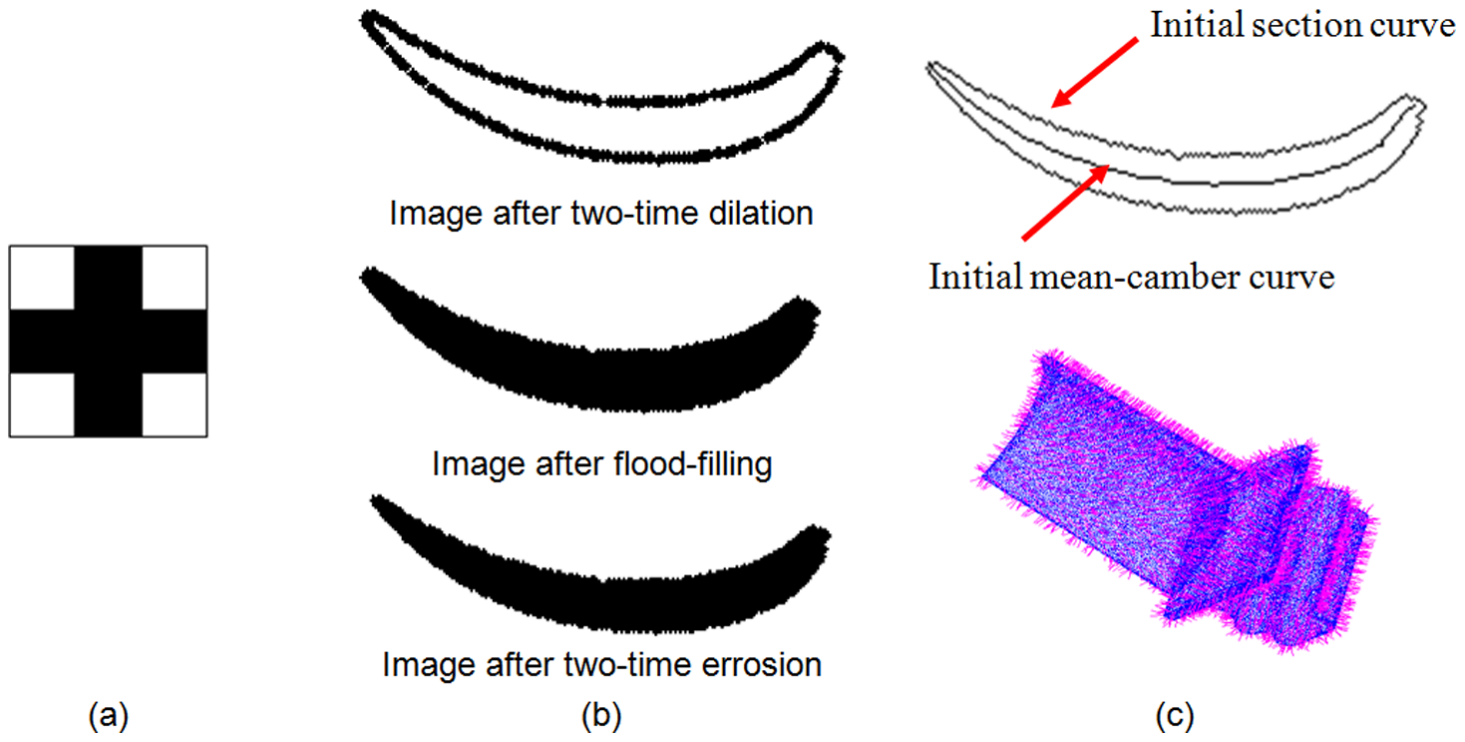
Mathematical morphology operation of a binary image from the blade model. (a) “+” structure element, (b) MM operation, (c) initial curves and normal vector.

Fig. 3(a) presents a point-sampled turbine surface, where section planes that are parallel to the reference plane are used to intercept the point cloud, and Fig. 3(b) shows the 2D points in one section plane. It is difficult to obtain the real intersection points between the section planes and point-sampled surface. In our implementation, the section plane 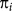 (Fig. 3(c)) is set from the reference plane with a relative distance 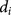 (which is determined based on the required number of blade inspections), and all points between planes 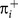 and 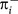 are projected onto plane 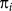. The MM operation, which includes dilation, flood filling and erosion, is implemented toward well-ordered points in Fig. 4(b). As shown in Fig. 4(c), an initial profile curve in one section plane can be generated by linking the well-ordered points after erosion, an initial mean-camber curve is rapidly generated by extracting the morphological skeleton from the well-ordered points, and the normal vector at each point is obtained by computing the gradient vector field of the binary image [28]. MM is advantageous because it can reduce the effect of the measured defects, such as holes, uneven density and miss-registration, and confirm the oriented normal information of the measured points. In the following section, the obtained initial curves and normal vector are used to implement the section curve reconstruction and parameter extraction.

### 2.2 Point cloud smoothing by energy minimization

The initial section curve in Fig. 4(c) is not smooth (folding line) because the position of the measured points are affected by the dilation/erosion operation and measuring noise. Assume that 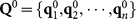 and 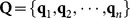 denote the measured points in one section plane before and after smoothing, respectively, and that 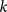 denotes the section curve curvature. Then, the strain energy 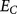 of a curve 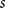 is
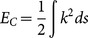
(4)


The curvature of a discrete point 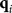 can be approximated by
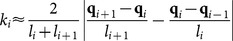
(5)where 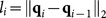. Because 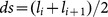, (4) can be represented by
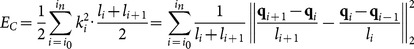
(6)


According to Zhu's work [29], the spring energy 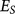 between point 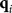 and point 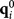 is 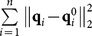. Then, the total energy function of point cloud smoothing is defined by
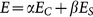
(7)where 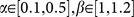. The strain energy 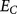 expresses the relationship between two adjacent points and is used to control the fairness of adjacent points 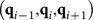. The spring energy 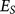 is used to control the distance deviation between unsmoothed point 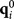 and smoothed point 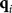, which avoids a major change in 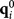.

1. If point set 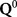 is located in an unclosed curve, 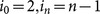;
2. If point set 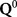 is located in a closed curve, 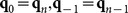 and 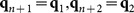, then 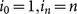.

To minimize the energy function 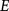 in (7), one differentiates energy 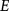 on variable 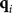; then,
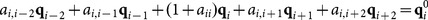
(8)


The coefficients in (8) are
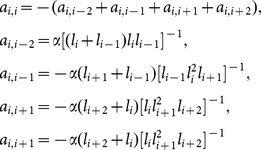



There are 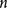 equations with 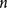 variables 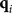, and the solution is unique. However, if 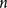 is high, the calculation and storage of equations will consume large amounts of memory space. In this paper, an iterative solving method is used to calculate the smoothed points in 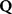. For a smoothed point 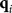, there exists
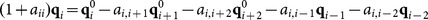
(9)


Therefore, during each iteration, there is

1. For an unclosed curve
  - The first smoothed point: 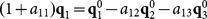

  - The second smoothed point: 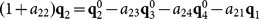

  - The (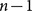)th smoothed point: 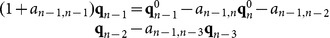

  - The 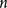
th smoothed point: 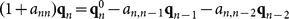

2. For a closed curve (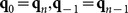 and 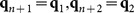)
  - The first smoothed point: 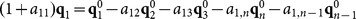

  - The second smoothed point: 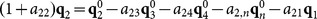

  - The (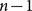)th smoothed point: 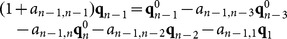

  - The 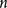
th smoothed point: 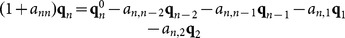

Because the measured points in the section plane are derived from a closed line, the second strategy above is used to iteratively calculate the smoothed points 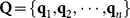.

### Curve reconstruction by distance minimization

Using the smoothed point 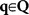, a cubic B-spline 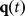 is constructed by
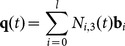
(10)where 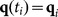 and 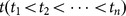 is a sequence of real numbers. The symbol 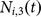 denotes the basis function of the B-spline, and 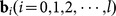 denotes the control points of the B-spline. The calculation of the curve 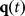 requires the interception of planar points, a dilation/erosion operation and energy-minimization smoothing. In particular, in the interception process, two section planes with a width of 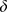 are used to surround the initially measured points from the blade model, and the surrounded points are subsequently projected onto a section plane. The surround points may not be real “intersection points” in the section plane, and the curve 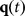 in (10) is only a fitting curve that passes through the smoothed points in Section 3. Therefore, the curve 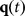 is considered an initial section curve and is iteratively updated to approximate the measured points in 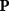.

**Figure 5:**
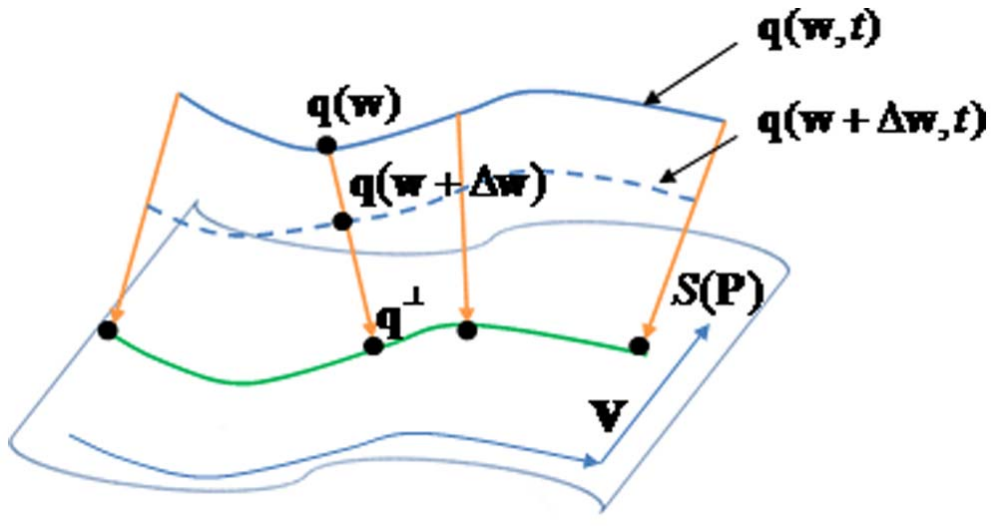
Point 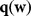 moves to the position of 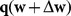 when there is a perpendicular 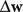.

In Fig. 5, assume that 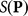 denotes the design surface of the blade and 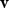 denotes the normal direction perpendicular to the section plane; for 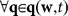, the foot projecting point 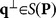 with respect to 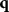 is calculated. The design surface 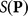 is unknown, and the foot point 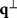 can be approximately replaced by the closest point of 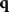 in 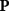. Then, the directed distance function between point 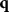 and 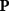 is defined by
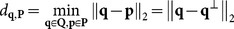
(11)


For point 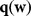, 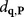 is a distance function with variable 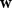. Ideally, if 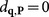, point 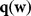 is located at 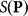. When a differential perturbation with respect to 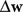 appears, 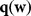 becomes 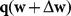. Assume that the foot projecting point of 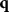 is still 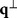; then,
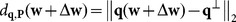
(12)


The first-order Taylor expansion at 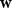 is
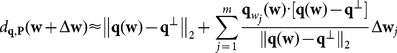
(13)where 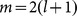 and 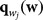 denotes the first-order derivative with respect to the variable 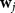. Then, 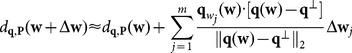
(14)


To simplify the calculation process, one scatters the initial B-spline curve into 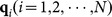 if the distance between two adjacent points is not larger than 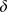. Then, the problem of constructing the section curve 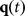 of the point-sampled blade surface becomes
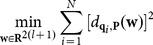
(15)


According to (14), the nonlinear least-square problem of (15) corresponds to
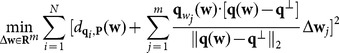
(16)


To limit the excessive offset of the variable 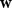, the coefficient 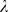 is added to the constraint. The final optimization model is
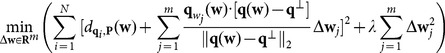
(17)


The implementation steps are as follows:

1. Perform MM and energy minimization to obtain a group of smoothed points and generate an initial cubic B-spline curve 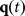;
2. Scatter 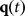 into 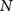 points 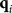 and search for the foot-projecting point 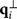 of 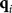 in 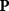;
3. Minimize (17) and obtain the offset 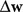 of the control variable during each iteration, where the value of 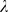 is set as 4;
4. Update the control variable by (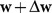) and obtain an updated curve 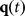;
5. Repeat steps (2)-(4) until 
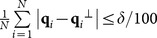
 or the number of iterations exceeds 20.

### Mean-camber curve extraction of the point-sampled blade surface

An aviation blade has many geometric parameters, such as the mean-camber curve, maximum gauge, leading/trailing edge, chord length, chord inclination, torsion resistance, and tortuosity. The manufacturing accuracy is important in the service performance of the blade. The mean-camber curve is a continuous curve of in-circle centers, where the maximum gauge corresponds to the maximal inscribed circle. The mean-camber curve is an important design basis, and a marginal shift may decrease the aviation aerodynamic performance considerably. Calculation and inspection of the mean-camber curve are vital to blade manufacturing. In the following, we introduce the method to extract the mean-camber curve.

**Figure 6:**
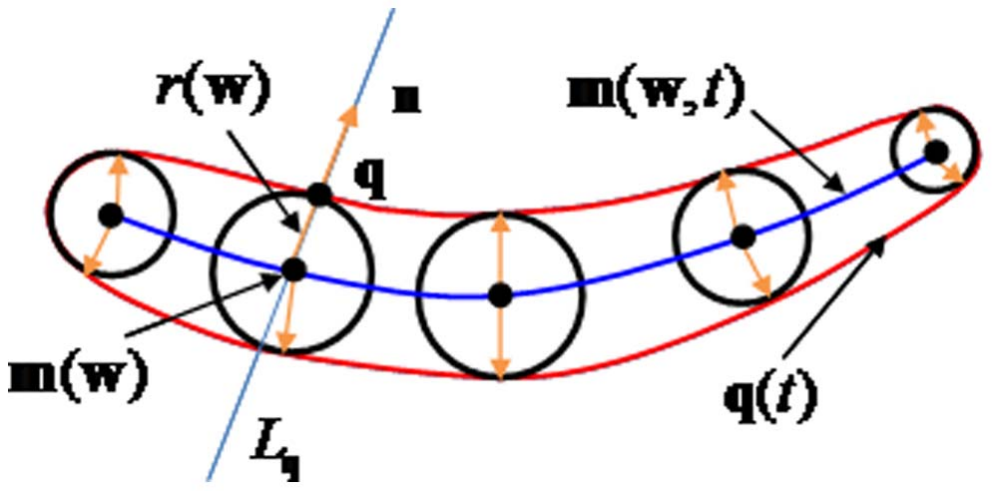
Calculation process of the mean-camber curve.

A group of point set 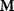 from the mean-camber curve is initially generated using the skeleton MM implementation presented in Section 2. For 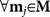, the two nearest points 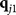 and 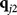 are calculated in the suction and pressure surfaces of the constructed curve 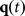, respectively. Assume that 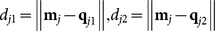, and define 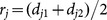 as the inscribed circle radius at point 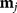. Then, 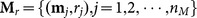 constitutes a group of new point sets and yields an envelope curve 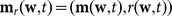 in Fig. 6, where 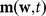 denotes the mean-camber curve and 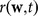 denotes the radius of the inscribed circle. For the convenience of calculation, the control point set is expressed as a column vector 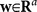 below.

First, the reconstructed curve 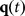 is scattered into 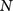 points. For 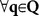, the normal vector is defined as 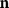, and a straight line 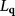 is generated using point 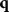 and vector 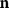. The intersection point between line 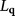 and the mean-camber curve is 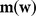; then, the directed distance from point 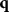 to the envelope curve 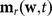 is
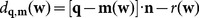
(18)


When a differential perturbation with respect to 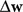 appears, 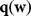 becomes 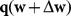, and the first-order Taylor expansions at 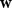 are
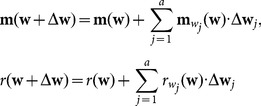
(19)where 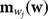 and 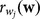 denote the first-order derivatives with respect to the variable 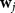. Because
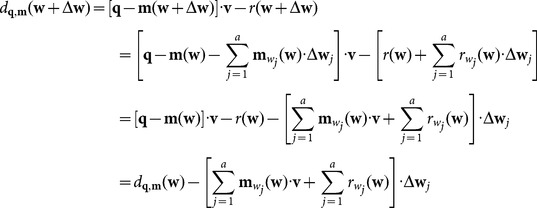
(20)


The first-order differential increment of 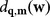 is
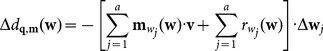
(21)


Therefore, the calculation process of the mean-camber curve becomes a nonlinear least-squares problem
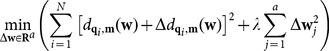
(22)


The calculation error of the mean-camber curve is defined by
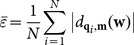
(23)


The implementation steps are as follows:

1. Scatter 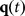 into 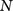 points 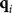 (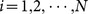) and maintain the dense points in the regions of the leading and trailing edges;
2. For each point 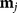, calculate its inscribed circle radius 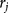 and obtain a new point set 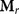. A cubic B-spline curve is used to construct an envelope curve 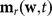;
3. Calculate the offset 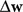 of the control point set during each iteration according to (22);
4. Apply the calculated 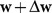 and update the envelope curve 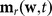;
5. Calculate the distance 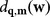 between 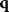 and the curve 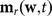 using (18);
6. Repeat steps (2)–(5) until 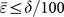 or the number of iterations exceeds 20.

## Experiments and Analysis

**Figure 7:**
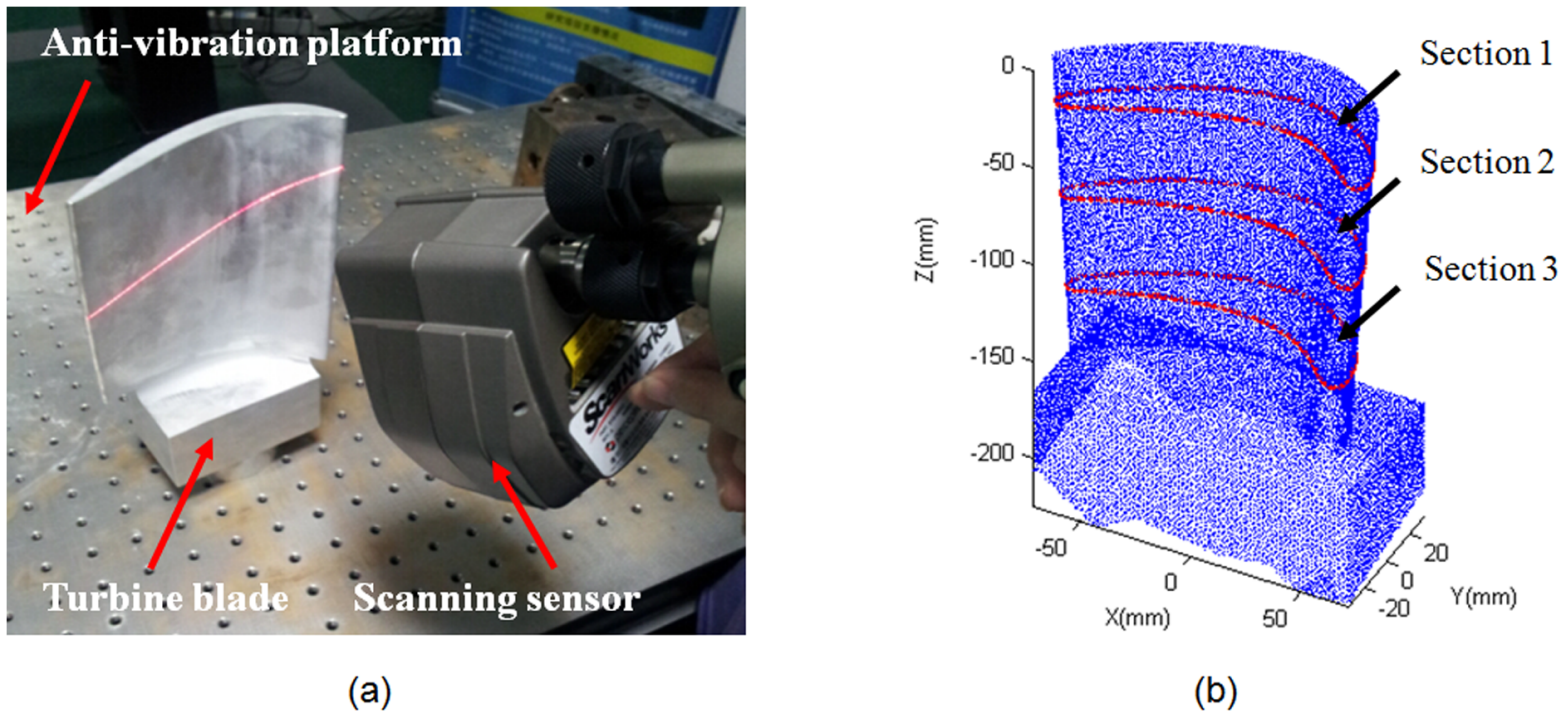
Scanning a turbine blade and intercepting its point cloud. (a) Turbine blade and scanning sensor, (b) Section planes and obtained measured points.

In Fig. 7(a), a turbine blade (150 mm×100 mm×40 m) is machined for the experimental analysis using a 5-axis Mikron CNC machine (UCP 800 Duro). The blade is placed on an anti-vibration platform and scanned using a Hexagon laser-scanning equipment (Infinite SC 2.4 m). The laser-scanning equipment has 6 rotational degrees of freedom and can scan the integral blade in one coordinate system without moving the blade or scanning equipment. The data scale of the obtained point cloud is 358,748, and the sampling space of the measured points after uniform simplification is 0.3 mm. Three section planes are used to intercept the point cloud and obtain three groups of 2D measured points (red color) in Fig. 7(b).

### Measured-point smoothing

**Figure 8:**
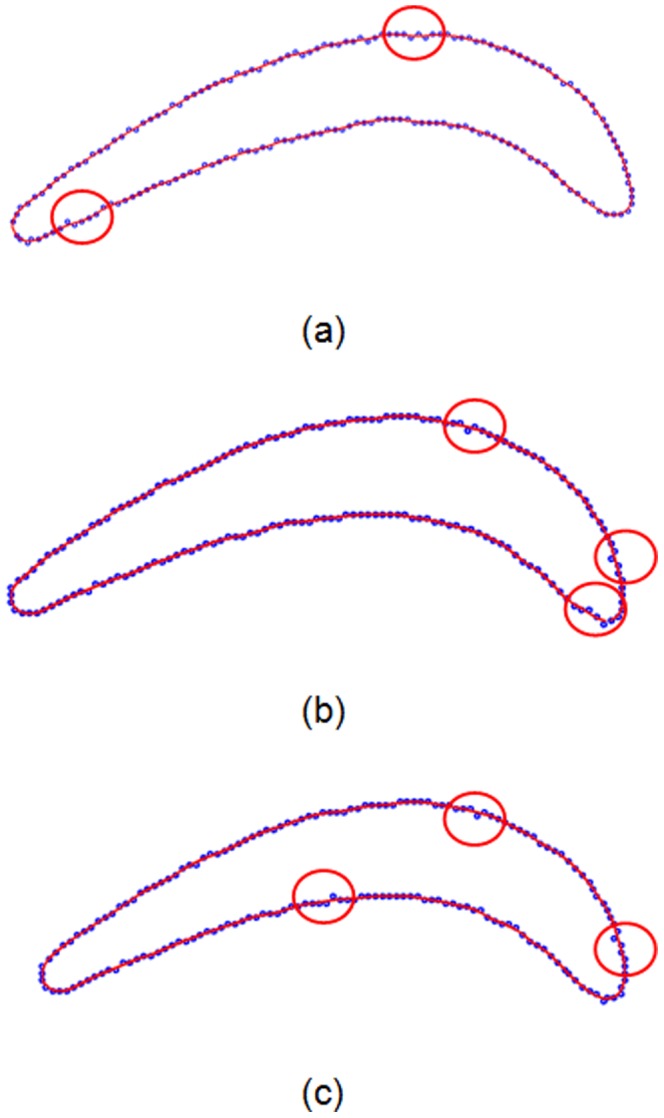
Measured points (blue) before smoothing and its folding line (violet) after smoothing in Section 1–3. Set 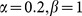, and perform 20 iterations.

**Figure 9:**
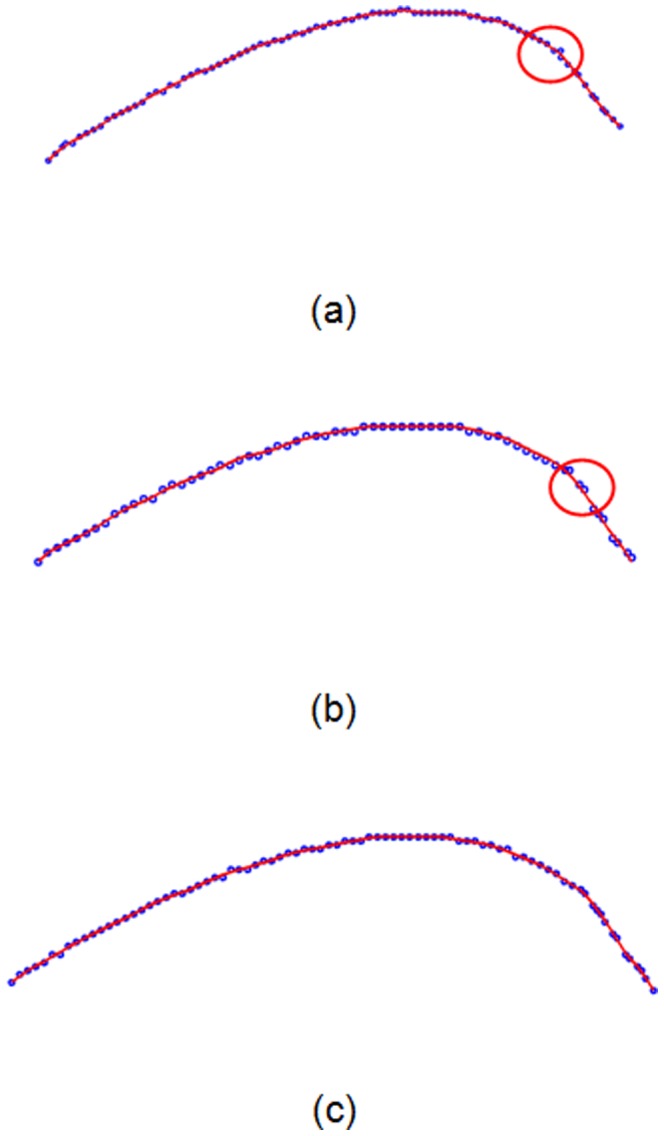
Mean-camber points (blue) from the morphological skeleton and its folding line (violet) after smoothing in Section 1–3.

**Figure 10:**
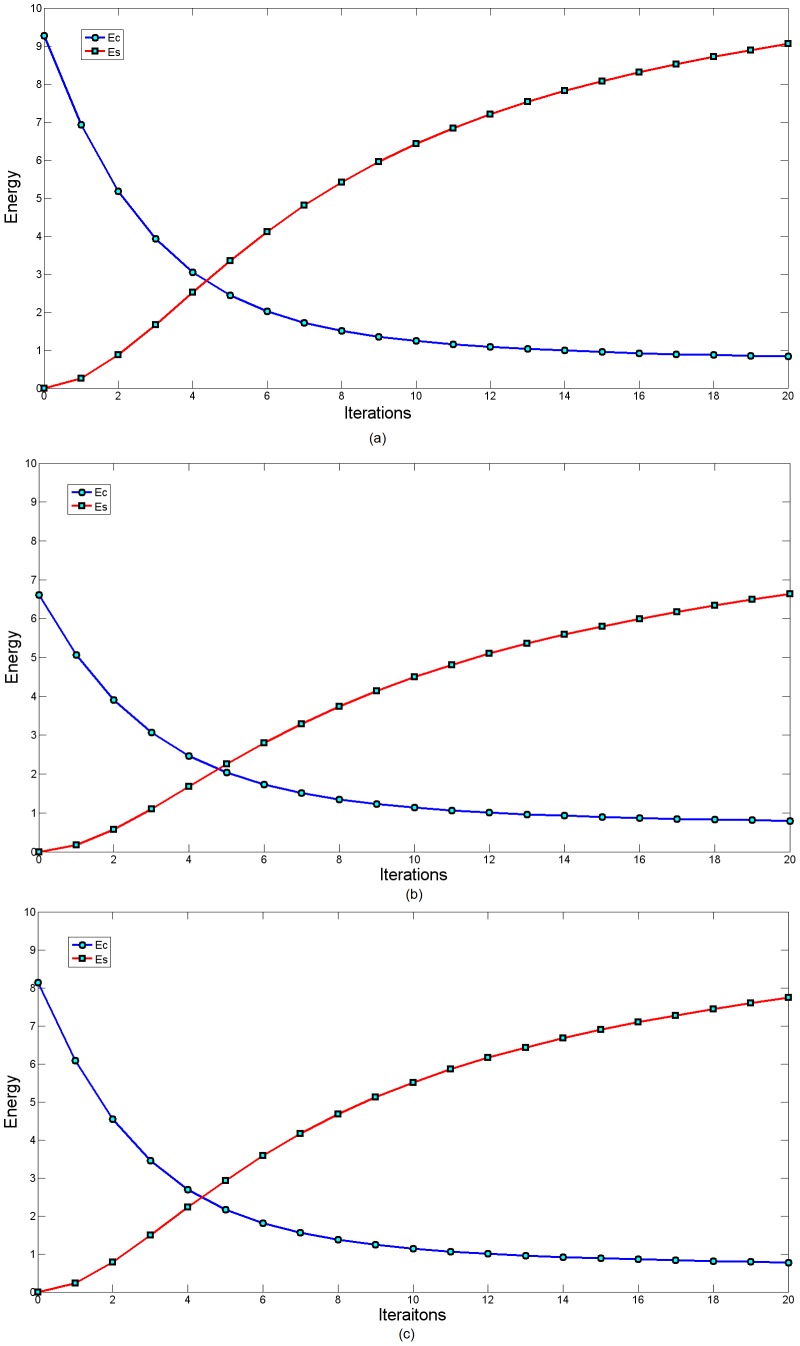
Energy variation of the section curves in Section 1–3 with an increasing number of iterations. Set 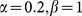, and perform 20 iterations.

**Figure 11:**
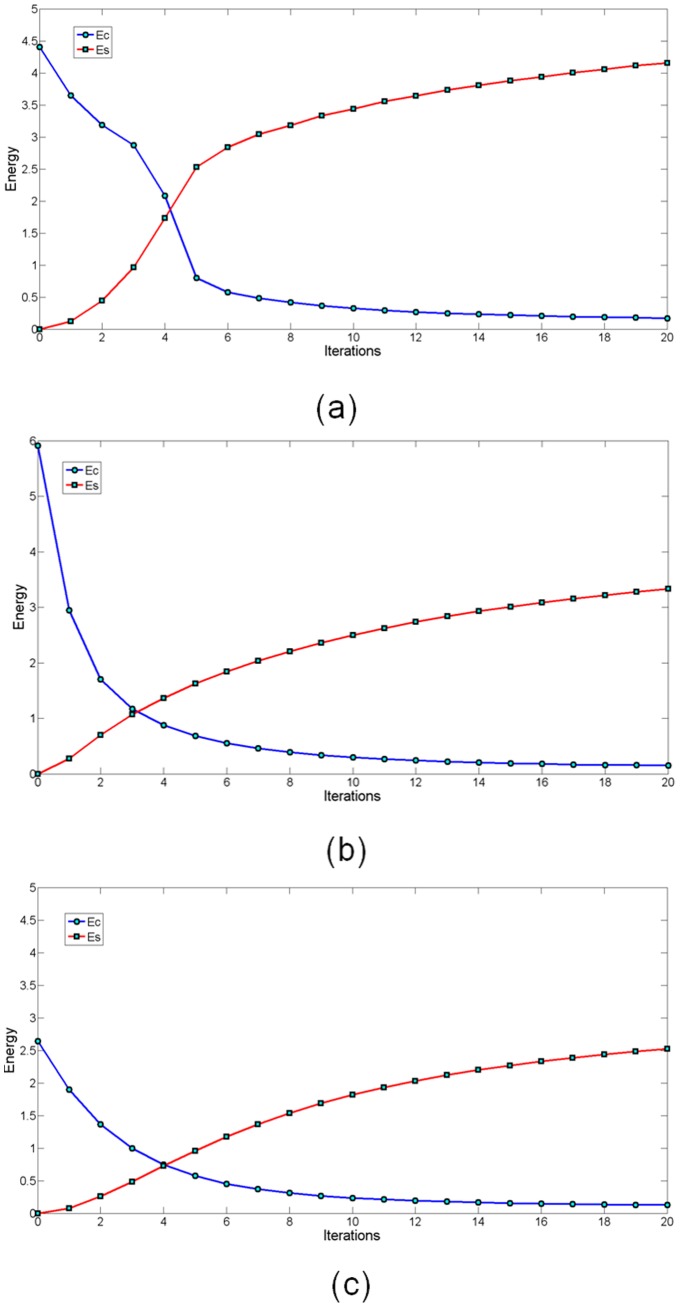
Energy variation of the mean-camber curves in Section 1–3 with an increasing iterations.

**Figure 12:**
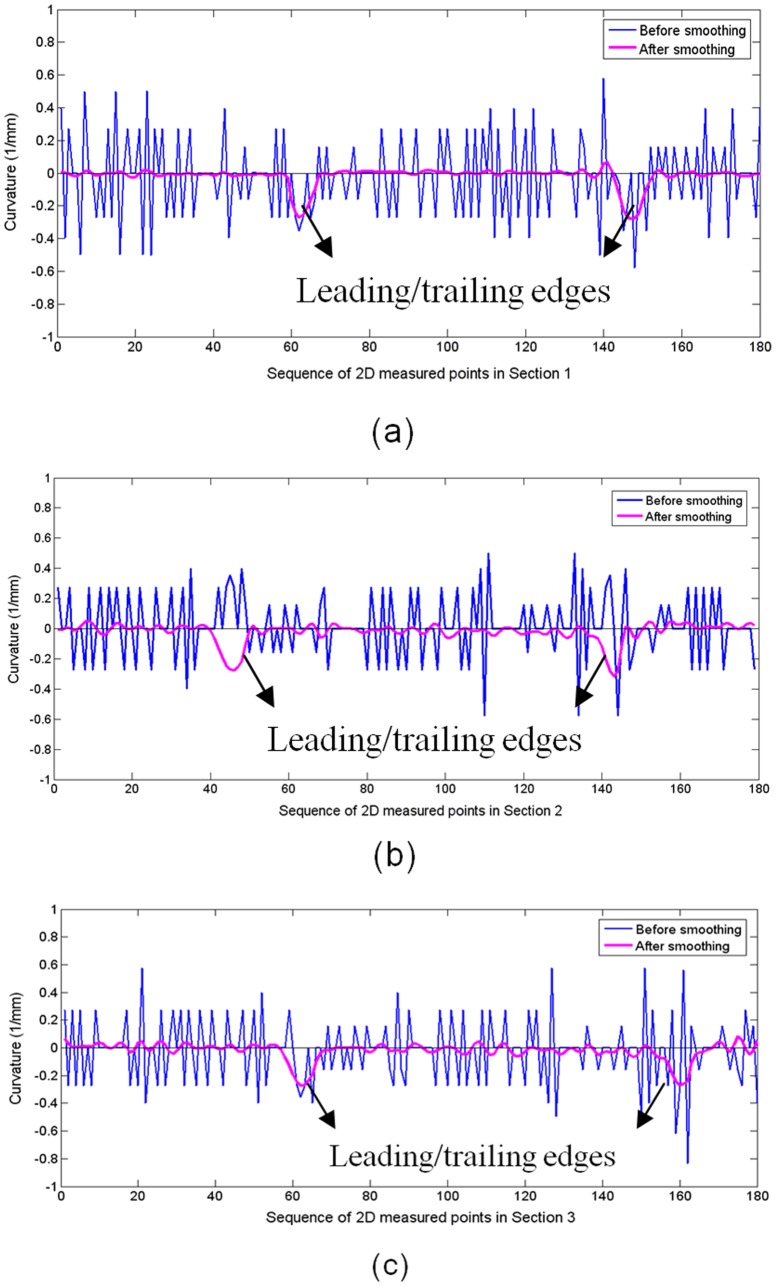
Variation in the curvature of the measured points after MM (blue) and the folding line (violet) that corresponds to Fig. 8.

**Figure 13:**
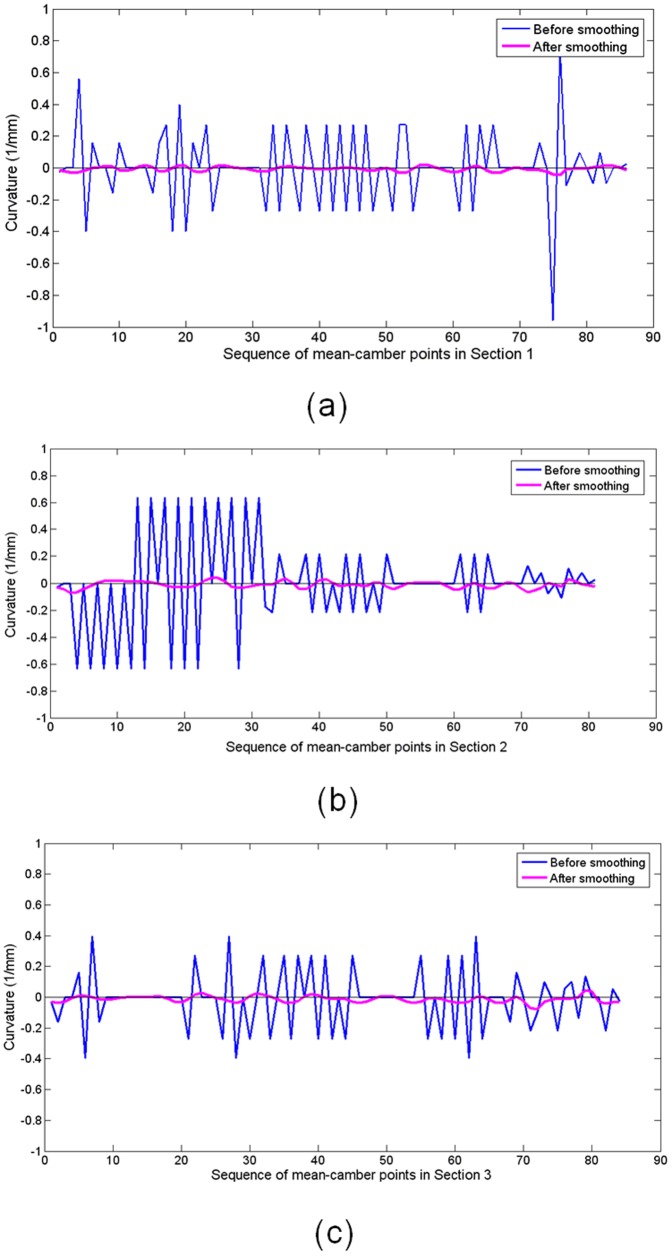
Variation in the curvature of the mean-camber points after MM (blue) and the folding line (violet) that corresponds to Fig. 9.

First, the strain energy in (4) and spring energy in (6) are calculated to construct a total energy function in (7), which is minimized to calculate the new position of the measured points. To obtain a stable strain energy of the 2D measured points, 20 iterations were run for convergence. The experimental results are shown in Figs. 8–13.

In Fig. 8, the measured points are marked with blue circles, and the measured points after smoothing are marked with red folding lines. To better graphically display the smoothing results, no more than 200 measured points are shown. The blue circles are uniformly distributed on two sides of the folding line. The same experimental results with respect to the mean-camber line are observed in Fig. 9. However, in some regions of the section curve, there is a large distance deviation (shown with red circles) between the measured points and folding line. The existing Gaussian noise is extremely high in these regions. The energy minimization in Section 3 suppresses the mutation of 2D measured points and maintains a fine fairness of the bolding line. The energy variations of the strain energy and spring energy are shown in Figs. 10 and 11, respectively. The strain energy, which controls the fairness of adjacent points, gradually decreases, and 15 iterations are sufficient to attain a stable value. From the 15^th^ iteration to the 20^th^ iteration, the strain energy slowly decreases, but the spring energy rapidly increases. Thus, the distance deviation between an unsmoothed point 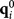 and a smoothed point 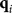 varies considerably to adapt to the fairness requirement. In the experiment, if the strain energy is approximately stable, it should stop the subsequent iterations to control the large movement of 2D measured points.

Figs. 12 and 13 present the variation in the curvature of the section curves and mean-camber curves, respectively. The blue polyline denotes the 2D measured points before smoothing, and the violet polyline denotes the folding line after smoothing. The variations in the curvature of the blue polyline are choppy, and the absolute value in some regions is relatively large. However, the curvature of the points smoothed via energy minimization nearly surrounds the X-axis, except for two high values at the leading/trailing edges. In addition, the plus-minus direction of curvature in the regions between the leading and trailing edges is not constant and does not fit with practical applications. The section curve is only represented by the folding line when energy-minimization smoothing is implemented, and it is not considered to maintain a direction consistency during each iteration. This problem can be solved using the distance minimization method.

### Section curve reconstruction

**Figure 14:**
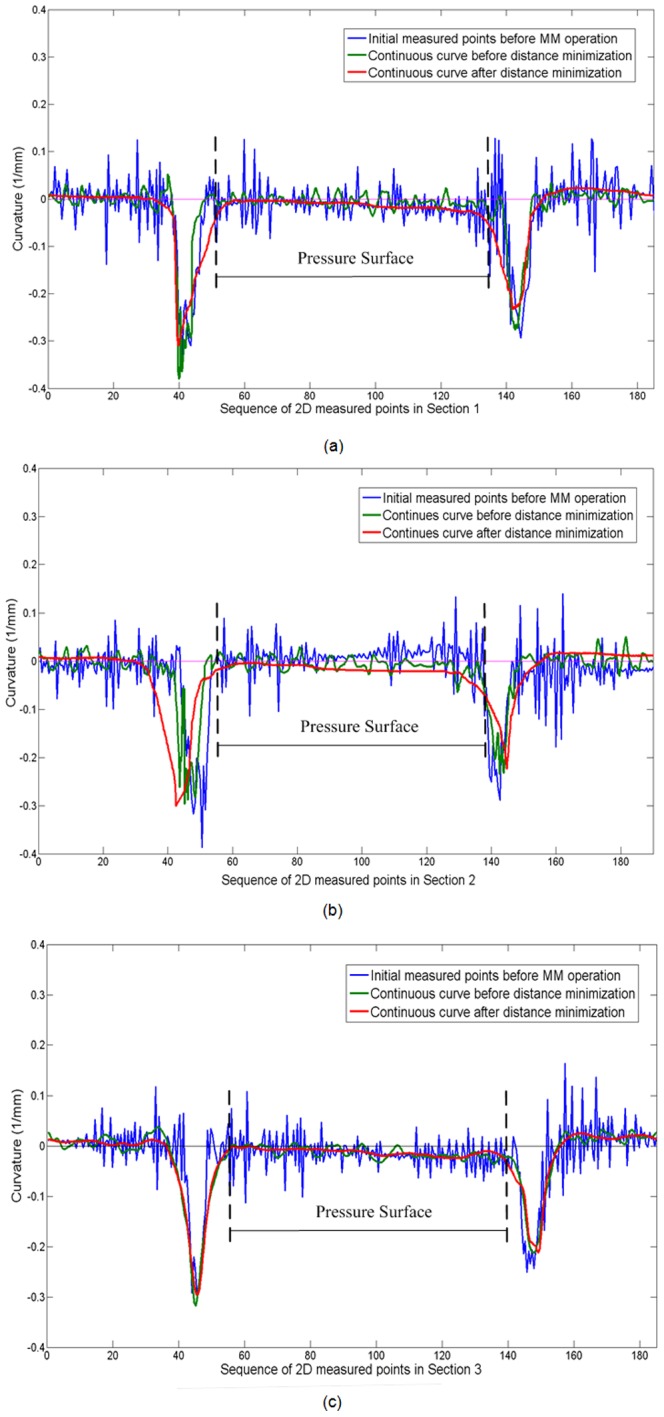
Curvature variation of the measured points (blue), continuous curve before distance function minimization (green) and continuous curve after distance function minimization (red).

The smoothed points in Section 1–3 are used to create three cubic B-spline curves according to (10). By minimizing the objective function in (17), the variations in the control points of the cubic B-spline curve are iteratively calculated and revised. In this experiment, 20 iterations are performed to obtain the optimal variable. The curvature values of the measured points before performing the MM operation (blue), the continuous B-spline curve before the distance minimization (jasper) and the continuous B-spline curve after the distance minimization (red) are obtained. The curvature variations in Section 1–3 are shown in Figs. 14 (a–c). To better display the curvature variation, the region of pressure surface is selected for further analysis. The curvature variation of the measured points oscillates and is unordered, and the curvature variation of the continuous curve before distance minimization (but after energy minimization) is stable around the X-axis. The main problem is that the plus-minus direction of curvature in the pressure region or suction region oscillates and does not maintain a consistent direction in the concave or convex surface. The continuous B-spline curve after distance minimization has the most realistic curvature variation. The curvature direction of the pressure surface is steadily negative and provides an increasing curvature value when approaching leading/trailing edges, which is consistent with practice.

**Figure 15:**
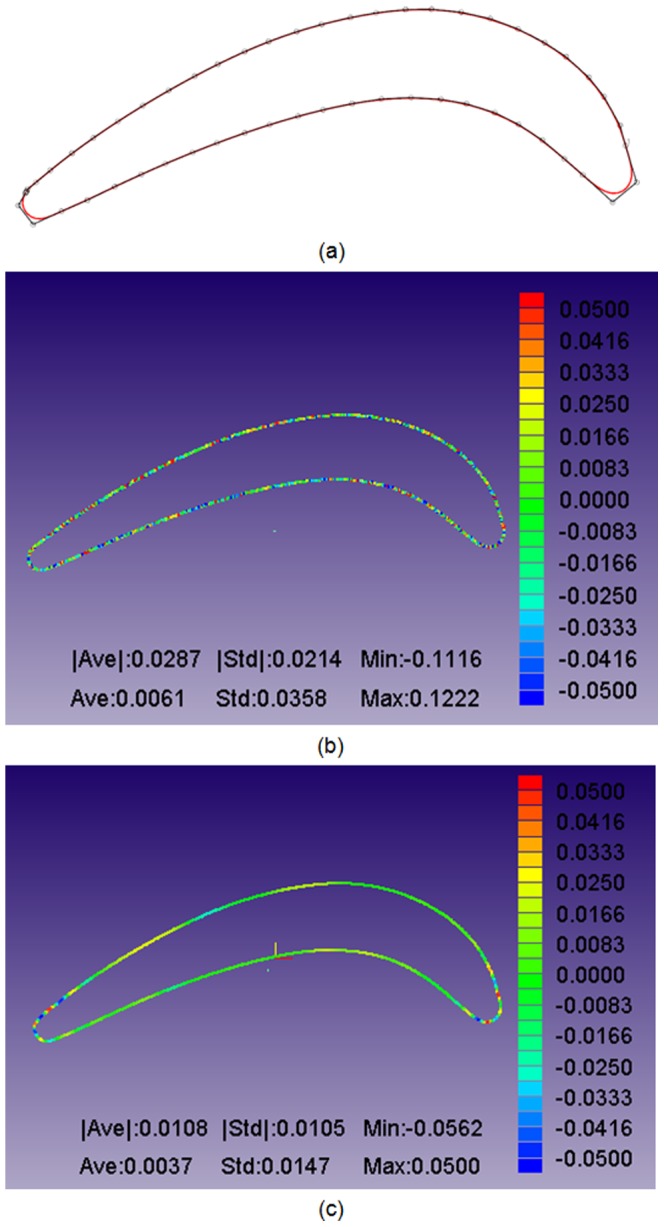
Curve reconstruction result in Section 1 after distance minimization: (a) reconstructed B-spline curve and its control points; (b) geometric errors between the measured points and B-spline curve; (c) geometric errors between the energy-smoothed points and B-spline curve. The displays of (b–c) are provided by iCloud3D software, which is developed by our team.

**Figure 16:**
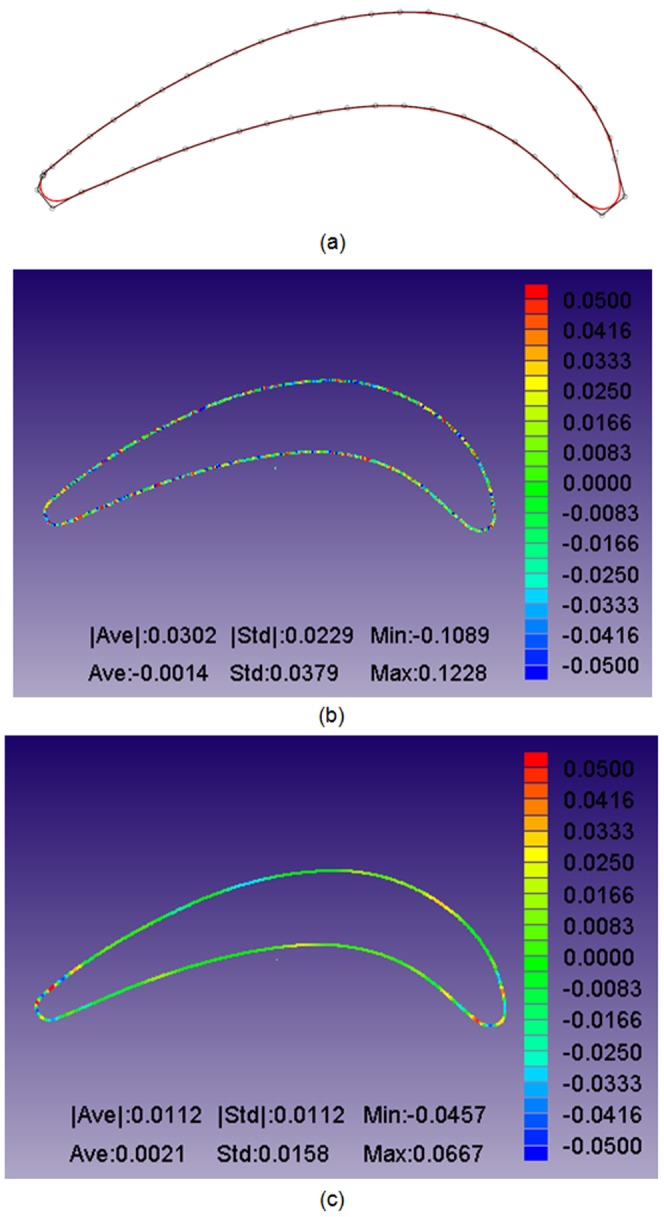
Curve reconstruction result in Section 2 after distance minimization: (a) reconstructed B-spline curve and its control points; (b) geometric errors between the measured points and B-spline curve; (c) geometric errors between the energy-smoothed points and B-spline curve. The displays of (b–c) are provided by iCloud3D software, which is developed by our team.

**Figure 17:**
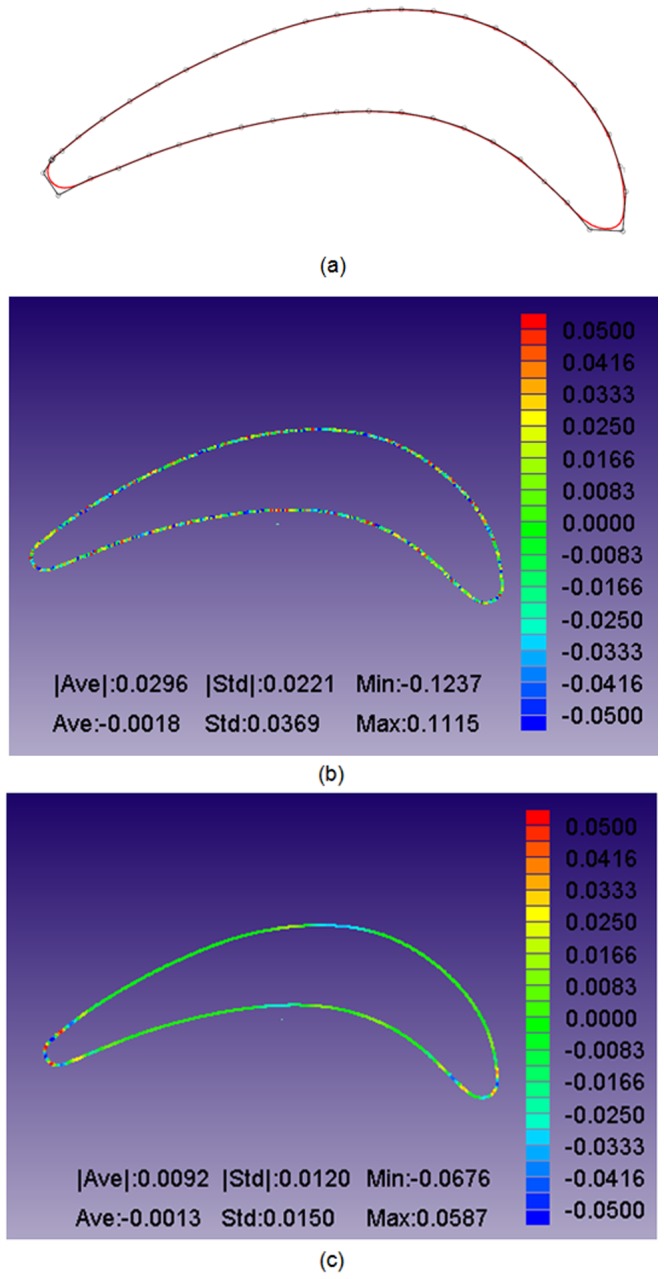
Curve reconstruction result in Section 3 after distance minimization: (a) reconstructed B-spline curve and its control points; (b) geometric errors between the measured points and B-spline curve; (c) geometric errors between the energy-smoothed points and B-spline curve. The displays of (b–c) are provided by iCloud3D software, which is developed by our team.

The constructed B-spline curves and control points after 20 iterations are shown in Figs. 15–17(a). To observe the constructed results in a quantifiable manner, the geometric errors of the average distance, distance standard error and mini/max error are calculated and shown in Figs. 15–17(b-c). Three continuous B-spline curves are obtained in Section 1–3. In Figs. 15–17(b), the value of the average distance 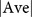 is approximately 0.03 mm. The error mainly originates from the measuring uncertainty of the Hexagon laser-scanning equipment. However, in Figs. 15–17(c), the value of 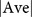 is reduced to approximately 0.01 mm, which benefits from the energy minimization in Section 3. The strain energy is used to smooth the measured points, and the spring energy is used to prevent a large deviation between an unsmoothed point and smoothed point, and provide a good initial value to iteratively construct a B-spline curve in Section 4.

### Mean-camber curve extraction

**Figure 18:**
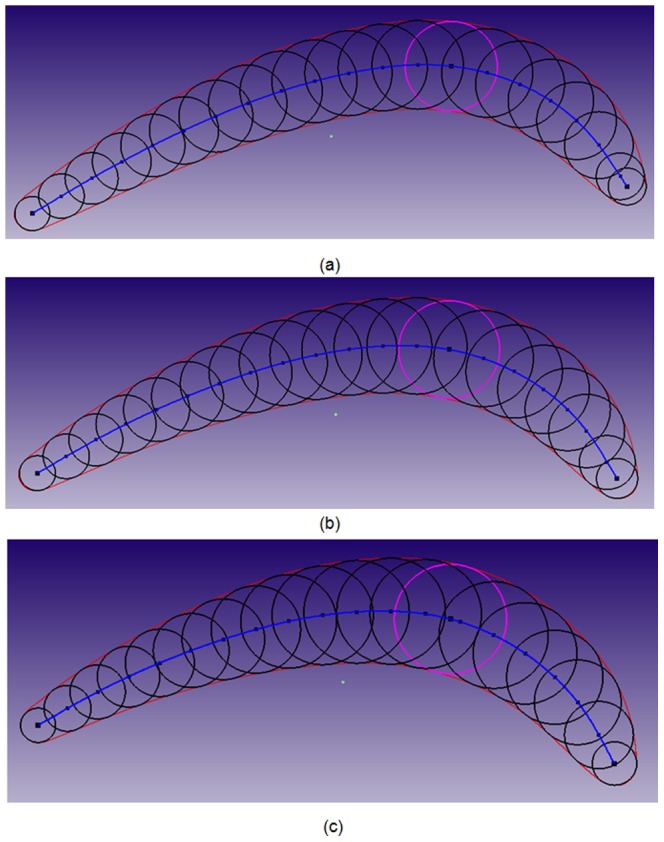
Graphical representation of the mean-camber curves and maximum gauge circles in Section 1–3, respectively. The displays of (a–c) are provided by iCloud3D software, which is developed by our team.

Finally, the implementation steps in Section 5 are performed to calculate the mean-camber curves of Section 1–3. The variations in the control points of each cubic B-spline curve are iteratively calculated and revised by minimizing the objective function in (22). Similarly, in this experiment, 20 iterations are performed to obtain the optimal value. The obtained mean-camber curves (blue), envelope circles (black) and maximum gauge circles (violet) are graphically shown in Fig. 18. To accurately calculate the maximum gauge circler, each B-spline curve of the mean-camber curve is scattered into 1,000 points, which are used to generate the envelope circles. To better display the generated envelope circles, approximately 50 points (from 1,000 points) and their corresponding circles are selected and shown in Fig. 18. In Figs. 18(a–c), the radii of the maximum gauge circles are 10.0011, 10.9182 and 11.8530 mm. Three mean-camber curves are successfully extracted, and the envelope circles are sufficiently close to the surrounding section curves.

**Figure 19:**
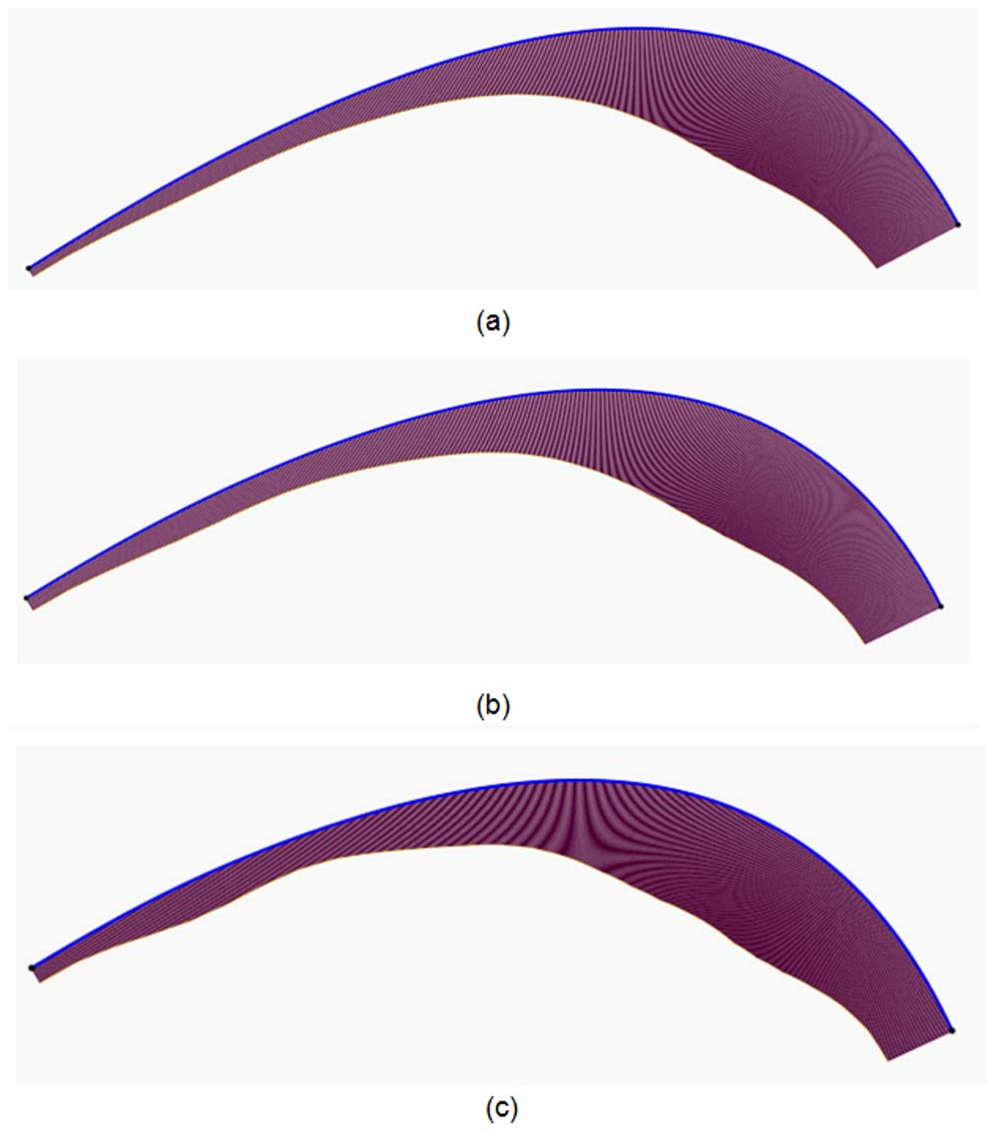
Graphical representation of the curvature value of the mean-camber curves in Section 1–3.

In addition, the curvature distributions of the three mean-camber curves are also calculated and displayed in Fig. 19. From the leading edge to the trailing edge, the curvature variation is rather consistent, which further demonstrates variable of parameter extraction.

## Conclusion

Rapid advancements in 3D scanning techniques have led to dense and accurate point clouds from real objects. Point cloud processing, curve approximation and parameter analysis based on 3D scanning have become a topic of considerable interest in blade manufacturing. This paper proposes a new method to address two common tasks in blade manufacturing: section curve reconstruction and mean-camber curve extraction. The main contributions of the proposed method include the following. First, the MM is expanded and applied to measured-point processing to restrain the effect of scanning defects. The results demonstrate that the MM implementation can also generate an ordered point sequence and provide an initial value for the curve approximation and parameter extraction. Second, the energy functions of the strain energy and spring energy are built to smooth the 2D measured points while preventing a large distance deviation between the unsmoothed and smoothed points. This implementation process is based on points and lines and is thus easy to implement. Third, a directed distance function from a measured point to its foot point or an envelope curve is defined and used to build a constrained nonlinear least-squares function. The purpose of this function is to iteratively approximate the cubic B-spline curve and extract the mean-camber curve using distance minimization. In addition, a turbine blade is machined and scanned to implement the experiments. The values of curvature variation, energy variation and approximation error are obtained and analyzed. The experimental results demonstrate the availability of the proposed method.

Curve reconstruction and parameter extraction are common problems in blade manufacturing, and the proposed method can find specific applications in the following fields. Future work will focus on practical applications of the proposed method.

1. **Aviation casting-blade finish inspection.** A turbine blade is typically short (300–600 mm), and a casting technique can be used to directly form its geometric shape. Measurements via a CMM are a common but inefficient inspection strategy in blade manufacturing. In practice, casting-blade finish inspection can be performed based on a 3D scanning method because there is nearly no reflective problem from the casting part. In this application, the calculation of the section curve and parameters is an important task.
2. **Large forging-blade allowance inspection.** A nuclear blade is rather long (600–2,000 mm). A general manufacturing process includes hammer forging, transformation straightening, milling and profile inspection. Mold wear and thermal stress releasing after forging typically result in uncertain blade transformation. Before milling, the uniformity of machining allowance in selected section planes should be inspected. The existing contact snap-gauge inspection based on naked eye/light transmission is not accurate and can be replaced by a 3D scanning method. In this application, the dislocation, mean-camber line and maximum gauge are three main parameters that must be calculated.
3. **Visual-guided robot grinding localization.** Robot grinding has the advantages of flexible contacting and wide-line machining and can lead to high surface roughness and quality consistency compared to traditional artificial grinding. One important task is to construct a relationship between the blade workpiece in the robot coordinate system and the grinding path in the design coordinate system. The workpiece can be scanned using a laser scanning sensor, and the obtained point cloud is used to register the design model. The registering results can be evaluated using the mean square error and section parameter error to determine whether the grinding allowance is satisfactory.

## References

[1] Hsu TH, Lai JY, Ueng WD, Hwang JZ (2005) An iterative coordinate setup algorithm for airfoil blades inspection. International Journal of Advanced Manufacturing Technology 26(7–8):797–807.

[2] Hsu TH, Lai JY, Ueng WD (2006) On the development of airfoil section inspection and analysis technique. International Journal of Advanced Manufacturing Technology 30(1–2):129–140.

[3] Chang HC, Lin AC (2005) Automatic inspection of turbine blades using a 3-axis CMM together with a 2-axis dividing head. International Journal of Advanced Manufacturing Technology 26(7–8):789–796.

[4] Makem Je, Ou HG. Armstrong (2012) A virtual inspection framework for precision manufacturing of aerofoil components. Computer Aided Design 44(9):858–874.

[5] Besl P, McKay H (1992) A method for registration of 3-D shapes. IEEE Transaction on Pattern Analysis and Machine Intelligence 14(2):239–256.

[6] Savio E, De Chiffre L, Schimitt R (2007) Metrology of freeform shaped parts. CIRP Annals- Manufacturing Technology 56(2):810–835.

[7] Heo EY, Kim DW, Lee JY, Kim KY (2008) Computer-aided measurement plan for an impeller on a coordinate measurement machine with a rotating and tilting probe. Robotics and Computer-Integrated Manufacturing 24(6):788–795.

[8] Li YQ, Ni J (2009) Constraints based nonrigid registration for 2D blade profile reconstruction in reverse engineering. ASME Journal of Computing and Information Science in Engineering 9(3):0310005 (9pp).

[9] http://aicon3d.com/applications/3d-scanner/quality-control/3d-quality-inspection-of-turbine-blades.html.

[10] Chen T, Du XM, Jia M, Song G (2010) Application of optical inspection and metrology in quality control for aircraft components. In: Proceedings of International Conference on Computer Engineering and Technology. Piscataway, USA, pp. 294–298

[11] Ravishankar S, Dutt HNV, Gurumoorthy B (2010) Automated inspection of aircraft parts using a modified ICP algorithm. International Journal of Advanced Manufacturing Technology 46(1–4):227–236.

[12] Pottmann H, Huang Q, Yang Y (2006) Geometry and convergence analysis of algorithms for registration of 3D shapes. International Journal of Computer Vision 67(3):277–296.

[13] Li WL, Yin ZP, Huang YA, Xiong YL (2011) Three-dimensional point-based registration algorithm based on adaptive distance function. IET Computer Vision 5(1):68–77.

[14] Yimaz O, Noble D, Gindy NNZ, Gao J (2005) A study of turbomachinery components machining and repairing methodologies. Aircraft Engineering and Aerospace Technology 77(6):455–466.

[15] Gao J, Chen X, Yilmaz O, Gindy N (2008) An integrated adaptive repair solution for complex aerospace components through geometry reconstruction. International Journal of Advanced Manufacturing Technology 36(11–12):1170–1179.

[16] Yilmaz O, Gindy N (2010) A repair and overhaul methodology for aero-engine components. Robotics and Computer-Integrated Manufacturing 26(2):190–201.

[17] Zheng JM, Zhong GL, Chen X (2006) Worn area modeling for automating the repair of turbine blades. International Journal of Advanced Manufacturing Technology 29(4):1062–1067.

[18] Berger U, Janssen R, Brinksmeier E (1999) Advanced mechatronic system for manufacturing and repair of turbine blades. In: Proceedings of Symposium on Information Control in Manufacturing. Kidlington, UK, pp. 295–300

[19] Rong Y, X JT, Sun YW (2014) A surface reconstruction strategy based on deformable template for repairing damaged turbine blades. Proceedings of the Institution of Mechanical Engineers, Part G: Journal of Aerospace Engineering 228(8):1–13.

[20] Zhang X, Kuhlenkotter B, Knueuper K (2005) An efficient method for solving the signorini problem in the simulation of free-form surfaces produced by belt grinding. International Journal of Machine Tools & Manufacture 45(6):641–648.

[21] Huang H, Gong ZM, Chen XQ, Zhou L (2003) SMART robotic system for 3D profile turbine vane airfoil repair. 21(4):275–283.

[22] Chen Q, Sun ZG, Zhang WZ, Gui ZC (2008) A robot for welding repair of hydraulic turbine blade. In: Proceedings of IEEE Conference on Robotics, Automation and Mechatronics, Piscataway, USA, pp. 155–159

[23] Gan ZX, Zhang H, Wang JJ (2007) Behavior-based intelligent robotic technologies in industrial applications. Lecture Notes in Control and Information Sciences 362 pp. 1–12.

[24] Ji K, Luan J, Liu CJ, Mu DL, Mu LH, et al (2014) A prospective study of breast dynamic morphological changes after dual-plane augmentation mammaplasty with 3D scanning technique. PLoS ONE 9(3):e93010. 24671190 10.1371/journal.pone.0093010 PMC3966867

[25] Almukhtar A, Ju XY, Khambay B, McDonald J, Ayoub A (2014) Comparison of the accuracy of voxel based registration and surface based registration for 3D assessment of surgical change following orthognathic surgery. PLoS ONE 9(4):e93402. 24695577 10.1371/journal.pone.0093402 PMC3973674

[26] Serra J (1982) Image analysis and mathematical morphology, London, UK: Academic Press

[27] Aptoula E, Lefevre S (2007) A comparative study on multivariate mathematical morphology. Pattern Recognition 40(11):2914–2929.

[28] Vizilter YV, Sidyakin SV, Rubis AY, Gorbatsevich V (2011) Skeleton-based morphological shape comparison. Pattern Recognition 21(2):237–360.

[29] Zhu XX (2000). Modeling technology of free-form curve and surface. Beijing, China: Chinese Scientific Publishing

